# Brain metastasis in head and neck cancer: an analysis of 201 cases

**DOI:** 10.3389/fonc.2026.1663296

**Published:** 2026-04-13

**Authors:** Junhui Yuan, Hao Li, Wei Du, Dairong Cao

**Affiliations:** 1 Department of Radiology, The First Affiliated Hospital of Fujian Medical University, Fuzhou, Fujian, China; 2 Department of Medical Imaging, The Affiliated Cancer Hospital of Zhengzhou University & Henan Cancer Hospital, Zhengzhou, Henan, China; 3 Department of Anesthesia, Wensu County People’s Hospital, WCPH, Akesu, China; 4 Department of Head Neck, The Affiliated Cancer Hospital of Zhengzhou University & Henan Cancer Hospital, Zhengzhou, Henan, China; 5 Department of Radiology, Fujian Key Laboratory of Precision Medicine for Cancer, The First Affiliated Hospital, Fujian Medical University, Fuzhou, Fujian, China; 6 Department of Radiology, National Regional Medical Center, Binhai Campus of the First Affiliated Hospital, Fujian Medical University, Fuzhou, Fujian, China

**Keywords:** brain metastasis, head and neck squamous cell carcinoma, hypopharynx, larynx, oral cavity, oropharynx

## Abstract

**Objective:**

To elucidate the clinicopathological characteristics and survival trajectories of patients with head and neck squamous cell carcinoma (HNSCC) who developed brain metastasis (BM).

**Methods:**

This retrospective analysis enrolled patients diagnosed with HNSCC and BM from three tertiary care institutions. Survival following the diagnosis of BM and the patterns of BM were examined.

**Results:**

A total of 201 HNSCC patients with BM were analyzed, revealing an incidence rate of 1.1%. The median interval from the initiation of treatment to the diagnosis of BM was determined to be 2.5 years. The distribution of intracranial metastases was as follows: one metastasis in 65 cases, two or three in 84 cases, and four or more in 52 patients. Brain metastases were predominantly localized to the supratentorial region in 130 patients, while involvement of both supratentorial and infratentorial regions occurred in 56 patients. The majority of patients reported significant discomfort. All patients succumbed within two years of the initial BM diagnosis, with the median survival time of 3 months. In Cox model analyses, patients receiving whole-brain radiotherapy or stereotactic radiosurgery alone or those undergoing surgical intervention in conjunction with post-operative radiotherapy exhibited a reduced mortality risk compared to those receiving no treatment, with hazard ratios of 0.72 (95% confidence interval: 0.53-0.98) and 0.64 (95% confidence interval: 0.43-0.95), respectively.

**Conclusion:**

Brain metastasis in patients with head and neck squamous cell carcinoma is a rare occurrence, often correlated with lung metastasis. Local therapy for brain metastases, primarily involving whole-brain radiotherapy, whether administered alone or alongside surgical interventions, was found to be associated with modest prolonged survival durations.

## Introduction

Head and neck squamous cell carcinoma (HNSCC) ranks as the seventh most prevalent malignancy among all solid tumors, encompassing neoplasms arising from the oral cavity, oropharynx, larynx, hypopharynx, and nasal cavity/paranasal sinuses (1). At the time of initial treatment, over half of these cases present with locally advanced disease, often attributable to undetected lymph node metastasis. Even among those managed with surgery-based systemic therapies, 10% to 15% may subsequently develop distant metastases, while brain metastasis (BM) remains a rarity, with an incidence of less than 1% (2).

The recursive partitioning analysis associated with the Radiation Therapy Oncology Group (RTOG) BM trial has elucidated that patients undergoing whole brain radiotherapy (WBRT) can be stratified into three distinct categories based on their clinical presentation, primary tumor control, and the existence of extracranial metastases (3). This stratification is of paramount importance for the design of clinical trials and for predicting patient outcomes. Various research groups have subsequently created analogous scoring systems to evaluate survival rates. Furthermore, findings indicate that the survival outcomes and prognostic factors following the diagnosis of BM may differ significantly according to the type of primary malignancy.

Although detailed specific prognostic factors for lung, breast, skin, colorectal, and kidney cancers have been thoroughly delineated (4–8), studies focusing on HNSCC remain relatively scarce. To the best of our knowledge, fewer than 200 HNSCC patients with BM have been documented (9–13), the majority of whom exhibit a dismal median survival ranging from two to eleven months. Due to the limited sample sizes, the significance of prognostic factors in this cohort could not be adequately assessed. Accordingly, our objective is to analyze the clinicopathological features and survival patterns of HNSCC patients with BM based on data from three large tertiary hospitals.

## Patients and methods

### Ethical approval

This study was approved by Zhengzhou University Institutional Research Committee, and written informed consent for medical research was obtained from all patients prior to initial treatment. All methods were performed in accordance with the Declaration of Helsinki.

### Study design

To fulfill our objective, a retrospective review of medical records from 18, 243 patients treated for HNSCC was conducted at The Affiliated Cancer Hospital of Zhengzhou University, The Affiliated People’s Hospital of Zhengzhou University, and The First Affiliated Hospital of Zhengzhou University from January 2000 to October 2024. Inclusion criteria comprised patients with a histologically confirmed diagnosis of primary HNSCC, a diagnosis of brain metastasis based on contrast-enhanced MRI or CT, and the availability of complete clinical, radiological, and follow-up data. Patients were excluded if they had an ambiguous primary tumor site; if the primary tumor was nasopharyngeal carcinoma, due to its distinct biological behavior and higher propensity for intracranial spread; if the intracranial disease was suspected to result from direct extension or perineural spread of the primary tumor; if there was a prior history of other malignancies or synchronous primary cancers; or if there was a lack of radiological confirmation of brain metastases. Data concerning demographics, pathology, treatment modalities, and follow-up information were meticulously extracted.

### Statistical analysis

The primary outcome of interest was overall survival following brain metastasis diagnosis (post-BM survival). This was defined as the duration from the date of BM diagnosis to the date of death from any cause. Predictors influencing post-BM survival were initially analyzed using the log-rank test. Variables significant in univariate analysis (p<0.05) along with key clinical covariates (including the number, maximum dimension, and location of brain metastases) were included in a multivariate Cox proportional hazards model to identify independent predictors.

Proportional Hazards Assumption: The proportional hazards (PH) assumption for the final multivariable Cox model was assessed. This was done by examining the scaled Schoenfeld residuals for each covariate and globally. A p-value > 0.05 for the correlation between the residuals and time was considered evidence that the PH assumption was not violated. No significant departures from the proportional hazards assumption were detected.

All statistical analyses were performed using R version 3.4.4, with a p-value of less than 0.05 deemed statistically significant.

## Results

### Baseline data

In total, a consecutive cohort of 201 patients with HNSCC and BM was enrolled, with an incidence of 1.1%. The cohort had a mean age of 65 ± 12 years and was predominantly male (125 males vs. 76 females). A significant majority had a history of smoking (156 patients, 77.6%). The primary HNSCC was most frequently located in the oral cavity (72 patients, 35.8%) and oropharynx (48 patients, 23.9%), followed by the larynx, hypopharynx, and nasal cavity/paranasal sinuses. The initial treatment for the primary tumor was primarily surgery-based (169 patients, 84.1%), while the remainder were treated with definitive chemoradiation (32 patients, 15.9%). At the time of BM diagnosis, most patients had a poor performance status (ECOG score ≥2 in 146 patients, 72.6%) and the majority had regional nodal disease (196 patients, 97.5%). Concurrent lung metastasis was common (157 patients, 78.1%), whereas only 20 patients (10.0%) presented with isolated BM (see **Table 1**).

**Table 1:** Baseline data of the 201 head and neck squamous cell carcinoma patients with brain metastasis.

Variable	Number
Age	
≤ 65	67
> 65	134
Sex	
Male	125
Female	76
Smoking history	
Yes	156
No	45
Initial treatment	
Surgery-based	169
Chemoradiation	32
Primary site	
Oral Cavity	72
Oropharynx	48
Larynx	38
Hypopharynx	27
Nasal cavity/paranasal sinuses	16
ECOG performance score	
0-1	55
≥2	146
Regional disease	
No	5
Yes	196
Extracranial distant metastases	
None	20
Lung	157
Bone	48
Liver	28
Other	31

Regarding systemic therapy, a clear distinction was observed between treatment administered before and after BM diagnosis. Prior to BM diagnosis, the majority (180/201, 89.6%) had not received immunotherapy or cetuximab. Twenty-one patients (10.4%) had received at least one line of such systemic therapy, which included cetuximab (n=14), anti-PD-1 immunotherapy (n=6), or both (n=1). Following the diagnosis of BM, 15 patients (7.5%) received post-BM systemic therapy with cetuximab or immunotherapy.

### Characteristics of BM

The interval between initial therapy and the diagnosis of BM ranged from 0.3 to 13.6 years, with a median duration of 2.5 years. The number of intracranial metastases was as follows: one metastasis in 65 cases, two or three in 84 cases, and four or more in 52 patients. The maximum dimension of BM lesions measured 20 ± 12 mm. Of note, BM was confined to the supratentorial region in 130 patients, while both supratentorial and infratentorial regions were involved in 56 patients. Almost all patients (n=188) reported discomfort. (**Table 2**).

**Table 2:** Characteristics and treatment of the brain metastasis (BM).

Primary and BM diagnosis interval (years)	Number
Range	0.3-13.6
Median	2.5
Number of intracranial metastases	
1	65
2-3	84
>3	52
Maximum dimension of BM (mm)	20 ± 12
Location of BM	
Supratentorial only	130
Infratentorial only	15
Both	56
BM symptom	
No	13
Yes	188
Treatment	
No	37
WBRT/SRS	139
Radiotherapy and surgery	25

WBRT, whole-brain radiotherapy; SRS, stereotactic radiosurgery.

The clinical presentation at BM diagnosis was highly symptomatic, with 188 patients (93.5%) reporting neurological symptoms. These symptoms most commonly included headache (n=78, 38.8%), motor deficits such as hemiparesis (n=62, 30.8%), and seizures (n=22, 10.9%). Other presenting symptoms included cognitive disturbances, ataxia, and speech difficulties (**Supplementary Table 1**). Only 13 patients (6.5%) were asymptomatic at the time of BM diagnosis.

Regarding treatment for brain metastases, the majority of patients who received local therapy were treated with whole-brain radiotherapy (WBRT; 133 patients, 66.2%). A smaller subset underwent stereotactic radiosurgery (SRS; 6 patients, 3.0%), and surgical resection followed by radiotherapy (RT, 25 patients, 12.4%) was performed in others. The remaining patients received best supportive care (37 patients, 18.4%).

### Prognosis

**Figure 1:**
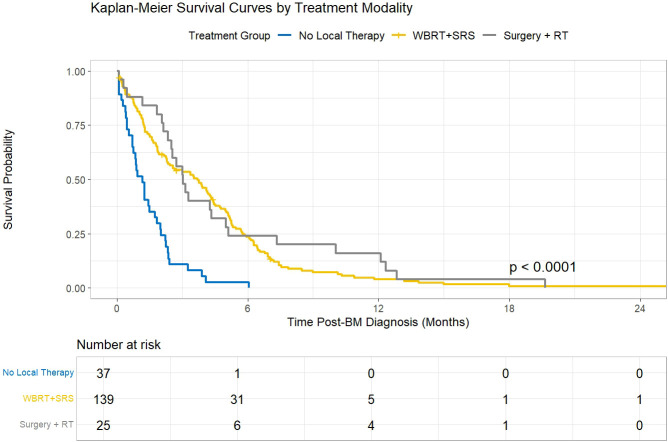
Kaplan-Meier survival analysis for head and neck squamous cell carcinoma patients with brain metastases, stratified by treatment modality. The number of patients at risk at specific time points is shown in the table below the graph. WBRT, whole-brain radiotherapy; SRS, stereotactic radiosurgery.

All patients succumbed within two years following the initial diagnosis of BM, with a median survival time estimated at 3 months. Survival outcomes stratified by treatment modality are presented in **Figure 1**. Kaplan-Meier analysis revealed significant differences in post-BM survival among the three groups (log-rank p < 0.001). Patients receiving any form of local therapy (WBRT/SRS or Surgery + RT) demonstrated markedly superior survival compared to those managed with best supportive care alone. The median survival was 1.5 months for the No Local Therapy group, 4.1 months for the WBRT/SRS cohort, and 4.6 months for the Surgery + RT group. Variables significant in univariate analysis (**Table 3**, p<0.05), along with clinically relevant covariates, were included in a multivariate Cox proportional hazards model to identify independent predictors of post-BM survival. As detailed in **Table 4**, the final model confirmed that older age (>65 years; HR = 1.31, 95% CI: 1.01-1.70, p=0.041), and a poor performance status (ECOG score ≥2; HR = 2.38, 95% CI: 1.53-3.70, p<0.001) were independently associated with an increased risk of mortality. Conversely, BM-directed treatment with WBRT or SRS alone (HR = 0.72, 95% CI: 0.53-0.98, p=0.036) or surgery combined with radiotherapy (HR = 0.64, 95% CI: 0.43-0.95, p=0.026) were independent predictors of prolonged survival compared to no local therapy. The proportional hazards assumption for the final model was tested and met (global p > 0.05). Other variables, including sex, primary tumor site, smoking history, initial treatment modality, number and size of brain metastases, and the presence of extracranial metastases, were not independently associated with post-BM survival in the multivariate model. (**Table 4**).

**Table 3:** Univariate Cox regression analysis for predictors of post-brain metastasis (BM) survival (n=201).

Variable	Category	HR (95% CI)	P
Age	≤65 years	Ref	
	>65 years	1.45 (1.10-1.91)	0.008
Sex	Male	Ref	
	Female	1.52 (1.10-2.10)	0.011
Primary Site	Oral Cavity/Oropharynx	Ref	
	Larynx/Hypopharynx/Nasal	1.14 (0.87-1.50)	0.327
Smoking History	Yes	Ref	
	No	0.93 (0.73-1.18)	0.536
ECOG Performance Score	0-1	Ref	
	≥2	2.85 (2.05-3.96)	<0.001
Number of Brain Metastases	1	Ref	
	2-3	1.18 (0.92-1.52)	0.195
	≥4	1.25 (0.90-1.73)	0.189
Maximum Dimension of BM	≤30 mm	Ref	
	>30 mm	1.28 (0.97-1.69)	0.090
Lung Metastasis	No	Ref	
	Yes	1.30 (0.98-1.72)	0.068
Liver Metastasis	No	Ref	
	Yes	1.35 (0.99-1.84)	0.057
Bone Metastasis	No	Ref	
	Yes	1.15 (0.87-1.52)	0.324
Number of Extracranial Metastatic Sites	0	Ref	
	1	1.10 (0.82-1.48)	0.525
	2	1.28 (0.94-1.74)	0.116
	≥3	1.42 (1.01-2.00)	0.045
Time from Primary Diagnosis to BM	<1 year	Ref	
	1-2 years	1.05 (0.78-1.41)	0.742
	2-5 years	0.94 (0.69-1.28)	0.691
	>5 years	0.79 (0.54-1.15)	0.215
Pre-BM Cetuximab/Immunotherapy	No	Ref	
	Yes	0.89 (0.57-1.39)	0.602
Post-BM Cetuximab/Immunotherapy	No	Ref	
	Yes	0.62 (0.40-0.96)	0.032
BM-Directed Treatment	No Treatment	Ref	
	WBRT/SRS	0.65 (0.50-0.85)	0.001
	Surgery + Radiotherapy	0.55 (0.40-0.76)	<0.001

WBRT, whole-brain radiotherapy; SRS, stereotactic radiosurgery.

**Table 4:** Multivariable Cox proportional hazards analysis (n=201).

Variable	Category	HR (95% CI)	P
Age	≤65 years	Ref	
	>65 years	1.31 (1.01-1.70)	0.041
ECOG Performance Score	0-1	Ref	
	≥2	2.38 (1.53-3.70)	<0.001
Number of Extracranial Metastatic Sites	0-1	Ref	
	≥2	1.29 (0.98-1.70)	0.069
Post-BM Cetuximab/Immunotherapy	No	Ref	
	Yes	0.69 (0.45-1.06)	0.089
BM-Directed Treatment	No Treatment	Ref	
	WBRT/SRS	0.72 (0.53-0.98)	0.036
	Surgery + Radiotherapy	0.64 (0.43-0.95)	0.026

Model adjusted for sex, primary site, and time to BM (all p>0.10). Likelihood ratio test χ²=58.7, df=7, p<0.001; concordance index=0.69.

## Discussion

This narrative elucidates the clinical pathology and treatment characteristics associated with BM in HNSCC. Our findings indicate that HNSCC accounts for approximately 1% of all cancer developments, underscoring the fact that BM occurrences are exceedingly rare. As anticipated, patients with BM exhibit disproportionately elevated rates of late primary tumor recurrence and regional relapse compared to the broader HNSCC population (14). Based on our clinical experience and existing literature, it is evident that BM in HNSCC typically originates and disseminates to distant sites, particularly the lungs, often presenting with symptomatic disease, poor performance status at diagnosis, and reduced survival rates. Patients receiving local therapy for brain metastases (primarily WBRT, with a small number receiving SRS) or surgical intervention demonstrated a statistically significant but modest survival benefit compared to those receiving solely supportive care.

With advancements in cancer treatment, while the incidence of BM appears to be rising alongside improved survival rates, the precise incidence remains elusive (15). Estimates derived from clinical and autopsy studies of cancer patients suggest an incidence range of 8.5% to 25%. The predominant sources of BM include malignancies and melanomas originating from the lungs and breasts. In contrast, BM resulting from HNSCC is considered relatively uncommon, with previously reported rates ranging from 0.03% to 1.3% (13), aligning with our observations. Nevertheless, a cohort study examining inoperable and advanced-stage patients revealed that BM was present in 5% of those with HNSCC, potentially reflecting a heightened prevalence in cases of advanced primary disease (11).

In our study, the majority of HNSCC cases with BM originated from the oral cavity and oropharynx. Notably, this differs from findings of the 2017 HNSCC BM study, where cases predominantly arose from the larynx (21%) and oral cavity (21%), with comparatively fewer cases from the oropharynx (11%) and salivary glands (5%) (16). However, our data are congruent with two other studies investigating BM in HNSCC, which similarly identified oropharyngeal squamous cell carcinoma as being predominant in various regions, including the throat and oral cavity, with infrequent cases arising from the lower pharynx (11, 14). In contrast, research conducted by Ghosh Laskar et al. (9) within the Indian population found that the most common primary sites were the throat (21%) and oral cavity (35%). These discrepancies may reflect variations in the prevalence of HNSCC within the general population, attributable to factors such as HPV infection, tobacco use, and alcohol consumption, which subsequently influence the relative proportions of OPSCC versus other head and neck subsites.

The incidence of HPV-positive oropharyngeal squamous cell carcinoma (OPSCC) has significantly increased, particularly associated with atypical transmission patterns and localization, notably in the brain. Prior researches revealed that their cases of OPSCC with brain metastasis were exclusively HPV-positive, comprising a total of 12 cases (17). Similarly, Bulut et al. (11) and Ruzevick et al. (18) observed advantageous proportions in their OPSCC BM cases, reporting ratios of 5:1 and 4:1 for HPV-positive to HPV-negative cases, respectively. Concurrently, Trosman et al. (19) documented 2 cases of HPV-positive and 2 cases of HPV-negative OPSCC with BM. However, p16 status was not routinely assessed in our patients.

In our study, the median interval to the occurrence of BM was found to be 2.5 years. These findings are consistent with previous results, who observed intervals of 17 and 19 months, respectively (9, 10). In contrast, Bulut et al. (11) and Ruzevick et al. (14) reported intervals of 26 and 36 months. This discrepancy may be attributed to the smaller sample sizes and the broad range of numerical values for this time interval, which spanned from 0.5 to 178.4 months in our investigation.

The median survival time for patients undergoing treatment for BM was recorded at 3 months. In comparison, patients receiving both radiation therapy and surgical intervention had a median survival time of 4.1 months, while those receiving only optimal supportive care had a median survival duration of 1.5 months. Notably, the 3-4 month survival rate observed in our study aligns with findings in other studies of non-head and neck-specific BM related to WBRT and supportive care lasting one month (9). A head and neck cancer-specific study by Ghosh Laskar et al. (9) found a median survival rate of 2 months following the diagnosis of BM, in contrast to Bulut et al. (11) (10.5 months) and Patel et al. (17) (16 months). This variation may stem from differences in patient populations and treatment modalities. In the study by Ghosh Laskar et al. (9), all patients received WBRT, while Patel et al. (16) treated patients exclusively with stereotactic radiosurgery (SRS). In our study, 66% of patients received WBRT, with 3% treated with SRS. Additionally, patients selected for SRS may generally possess superior performance status and better extracranial control, thus reducing the tumor burden within the cranium. Interestingly, patients receiving WBRT or SRS alone or surgery combined with radiotherapy demonstrated a reduced risk of death compared to those receiving no local therapy. However, it is important to interpret these hazard ratios with caution. While statistically significant, their confidence intervals narrowly cross the null value of 1.0, and the point estimates for the two active treatment strategies are remarkably similar. This pattern may indicate that our model is overfitted, a known risk in retrospective studies with a limited number of events relative to the number of covariates, or that it lacks the statistical power to precisely distinguish the effect size of different treatment modalities. Consequently, the most robust conclusion from our multivariate analysis is that any form of active local therapy (radiotherapy with or without surgery) is associated with a survival benefit compared to best supportive care, rather than asserting a definitive hierarchy between the specific treatment combinations.

Four favorable prognostic factors were identified in our univariate analysis: younger age (≤ 65 years), male gender, good performance status (ECOG score of 0-1), and BM treatment that included radiotherapy, with or without surgical intervention. Similar prognostic factors have been observed in non-HNSCC studies involving lung, breast, colorectal, and kidney cancers (20). As anticipated, performance status emerges as a generally reliable predictor of survival following BM across all cancer types. Age has been recognized as a predictive factor in previous studies, while gender has not consistently emerged as a predictor across lung, breast, and colorectal cancers. Intriguingly, common prognostic factors, such as the presence of extracranial metastases and the number of metastases, did not emerge as significant predictors in our study.

However, in our multivariate analysis, the treatment modality for brain metastases emerged as a significant predictor of survival. It is crucial to note that the local therapy received by the majority of our treated patients was WBRT. The observed survival benefit associated with local therapy must therefore be interpreted in the context of WBRT. The roles of WBRT and the increasingly utilized SRS are distinct, with SRS often reserved for patients with fewer metastases and better performance status. Recent evidence, including the QUARTZ trial, has questioned the survival benefit of WBRT over best supportive care in some patient populations with poor prognosis (21). The similar hazard ratios we observed for radiotherapy alone and surgery combined with radiotherapy may reflect our study’s limited power to distinguish between these modalities, especially given the very small proportion of patients (3%) who received SRS. Consequently, our findings primarily support the conclusion that active local therapy, predominantly WBRT in this cohort, is associated with a survival benefit compared to best supportive care in this selected group of HNSCC patients with BM, rather than defining a superior treatment strategy among the different local therapy options. Recent non-inferiority trials and retrospective analyses have indicated that patients receiving WBRT do not demonstrate a survival advantage over those receiving optimal supportive care (21, 22). Furthermore, the application of SRS has been expanding as a monotherapy for patients with a limited number of BM, with studies suggesting its efficacy even for those with up to 10 lesions. Moreover, research conducted by Furutani et al. (23) highlights that poorly differentiated brain tumors might benefit from SRS, especially in patients with significant intracranial tumor burden. The combination of radiotherapy and surgery has also shown substantial survival benefits compared to isolated treatment modalities. However, in our study, we noticed little difference in outcomes between the radiotherapy and surgical cohorts, potentially owing to the relatively low number of patients receiving both types of treatment in our institution.

The limitations of the present study warrant acknowledgment. Firstly, an inherent selection bias is present within the confines of a retrospective design. Secondly, the reliance on imaging analysis for confirming cases among many of our patients may compromise the overall accuracy of the study. Thirdly, the absence of data on HPV/p16 status limits the depth of our prognostic analysis and our ability to control for potential confounding factors known to influence HNSCC outcomes. Fourthly, the specific cause of death was not available for analysis, and it should be considered when interpreting the survival benefits associated with different treatment modalities.

In conclusion, brain metastases from HNSCC are rare and associated with poor prognosis. In this retrospective multicenter cohort, local intracranial therapies were associated with modest improvements in survival compared with supportive care alone. However, given the retrospective design and potential selection bias, these findings should be interpreted cautiously and require confirmation in prospective studies.

## Data Availability

The original contributions presented in the study are included in the article/**Supplementary Material**. Further inquiries can be directed to the corresponding author.
