# Supplementary material for: Brain metastasis in head and neck cancer: an analysis of 201 cases
Source: Front Oncol. 2026 Apr 13;16:1663296. doi: 10.3389/fonc.2026.1663296 (PMC13110937; doi:10.3389/fonc.2026.1663296)
Supplement: Supplementary file 1 [file Table1.doc]

****Supplementary Table 1. Detailed Neurological Symptoms at Brain Metastasis Diagnosis (n=201).****

| **Symptom Category** | **Specific Symptom** | **Number of Patients** | **Percentage (%)** |
| --- | --- | --- | --- |
| ****Headache**** | Generalized or localized | 78 | 38.8 |
| ****Motor Deficits**** | Hemiparesis / Limb weakness | 62 | 30.8 |
| ****Seizures**** | Focal or generalized | 22 | 10.9 |
| ****Cognitive/Mental**** | Confusion, memory loss, personality change | 18 | 9.0 |
| ****Cerebellar**** | Ataxia, gait disturbance, vertigo | 16 | 8.0 |
| ****Speech**** | Aphasia, dysarthria | 14 | 7.0 |
| ****Sensory Deficits**** | Paresthesia, numbness | 10 | 5.0 |
| ****Cranial Neuropathy**** | Diplopia, facial weakness, visual loss | 9 | 4.5 |
| ****Other**** | Nausea/vomiting, syncope | 11 | 5.5 |
| ****Asymptomatic**** | Incidental finding on imaging | 13 | 6.5 |
